# Schwannoma: A Rare Hoffa's Fat Pad Tumor

**DOI:** 10.1055/s-0039-1692996

**Published:** 2019-07-29

**Authors:** Jonathan Ruben Caballero Martel, Sara Estévez Sarmiento

**Affiliations:** 1 Department of Orthopedic Surgery and Trauma, Complejo Hospitalario Universitario Insular Materno Infantil de Gran Canaria, Spain

**Keywords:** schwannoma, intra-articular tumor, knee joint schwannoma

## Abstract

Hoffa's fat pad can be affected by a variety of tumors. Schwannomas are benign and typically solitary neoplasms of the peripheral nerve sheath; they are made up of the neoplastic Schwann cells and are usually located eccentrically. Malignant schwannomas are extremely uncommon. Here we report a case of an intra-articular schwannoma of the knee. A 54-year-old man presented with a painful lump in the medial aspect of the knee. Magnetic resonance imaging revealed a well-circumscribed intra-articular mass, which was later diagnosed as an intra-articular schwannoma based on biopsy findings.

The infrapatellar Hoffa's fat pad (HFP) is an intracapsular but extrasynovial structure located in the knee joint. Disorders of the HFP are a common cause of anterior knee pain. 1 Published studies have reported that tumors, categorized as either diffuse or solitary, can develop in the HFP. Solitary tumors are uncommon and mostly benign; among these tumors, schwannomas are extremely rare.

Benign schwannoma is the most common tumor of the peripheral nerves. Schwannomas are usually encapsulated, circumscribed, solitary, and located eccentrically and composed of the Schwann cells of the peripheral nerve sheath. Malignant transformation of schwannomas is extremely rare and accounts for less than 1% of all cases. 2 Schwannomas usually grow slowly and asymptomatically and are often detected incidentally. 3 In some cases, schwannomas may present symptoms, such as a lump, pain, or swelling.

Histologically, schwannomas have two distinctive areas of high and low cellularity called Antoni A and B areas, respectively. Schwannomas are usually entirely composed of Antoni A areas, where spindle cells are closely packed.

To the best of our knowledge, only one case of intra-articular schwannoma of the knee has been reported. Thus, in the current report, we aim to describe a rare and interesting case of an intra-articular schwannoma of the knee to increase its awareness in the orthopaedic community.

## Case Report

**Fig. 1:**
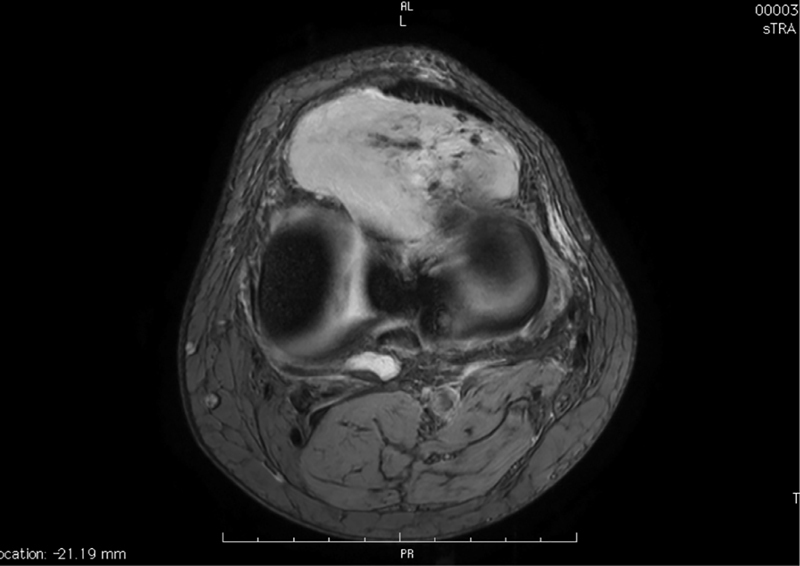
Coronal T2-weighted MRI showing a well-circumscribed schwannoma. MRI, magnetic resonance imaging.

**Fig. 2:**
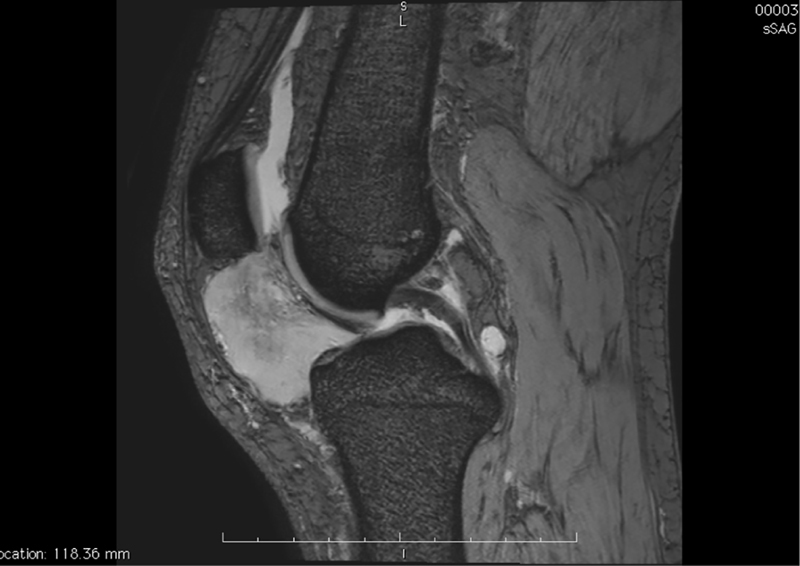
Sagittal T2-weighted MRI showing an infrapatellar schwannoma measuring 64 × 34 mm. MRI, magnetic resonance imaging.

**Fig. 3:**
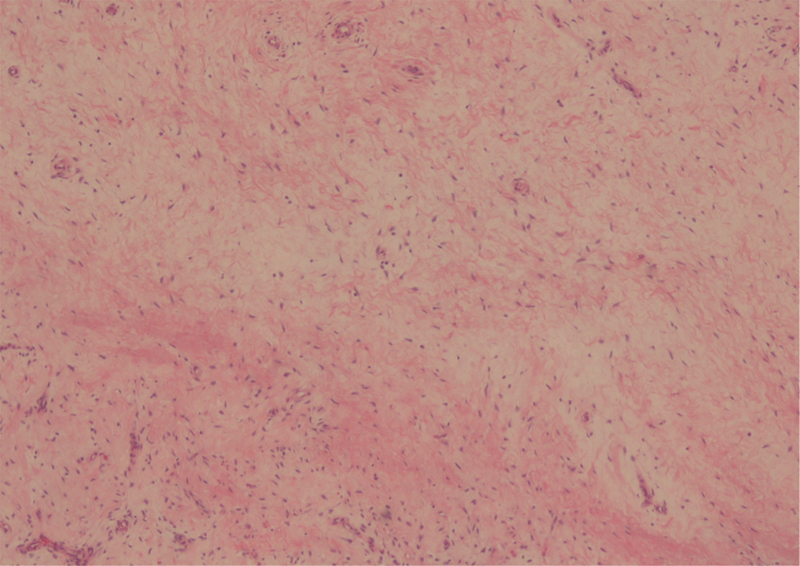
Intra-articular schwannoma. Fibrillar aspects and palisades of elongated spindle cells (hematoxylin and eosin staining; ×100).

**Fig. 4:**
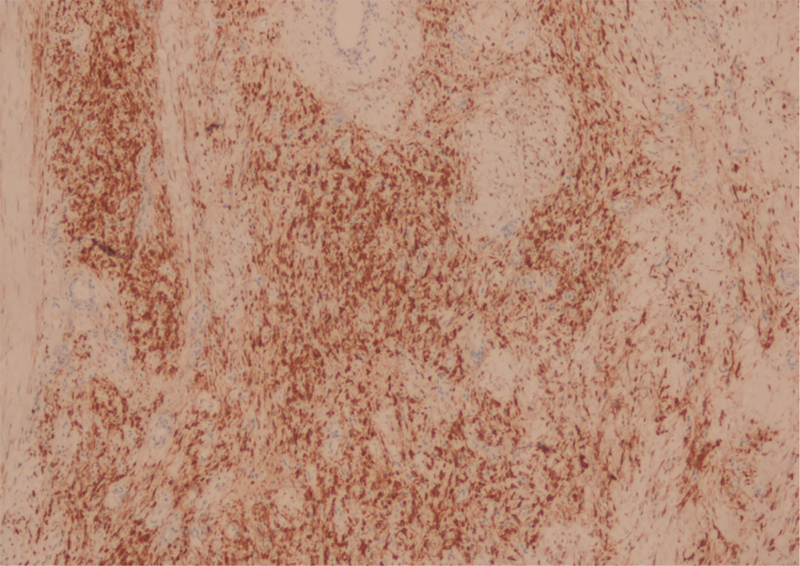
Intra-articular schwannoma. Immunostaining of S-100 protein demonstrates strong cytoplasmic immunoreactivity (×100).

**Fig. 5:**
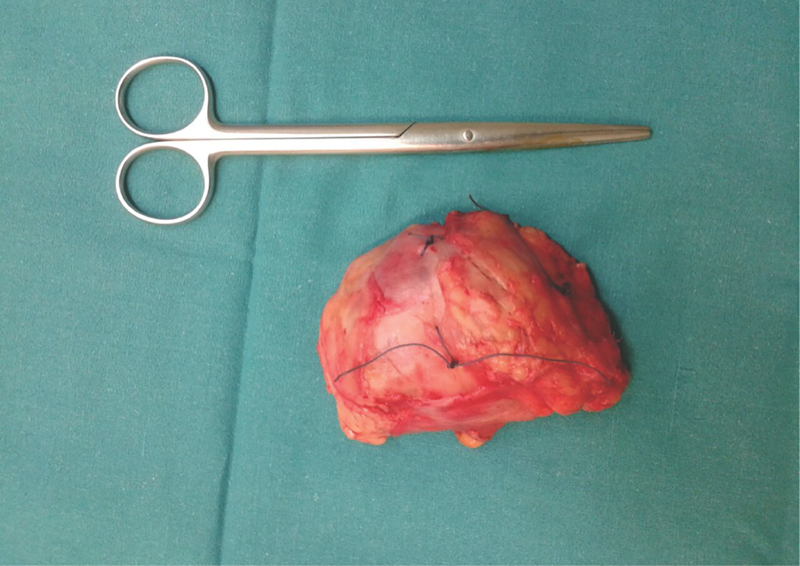
Surgical excision of the tumor: measurement of the resected specimen.

**Fig. 6:**
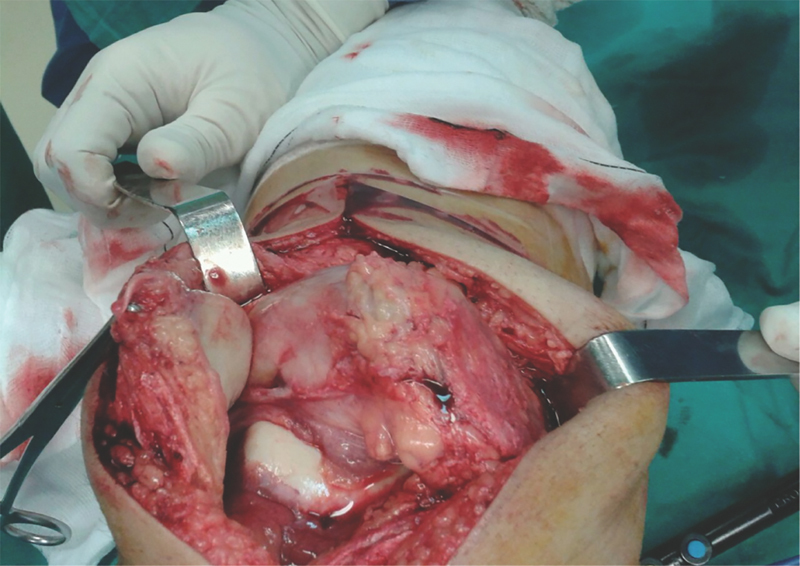
The medial parapatellar approach with tumoral exposure.

**Fig. 7:**
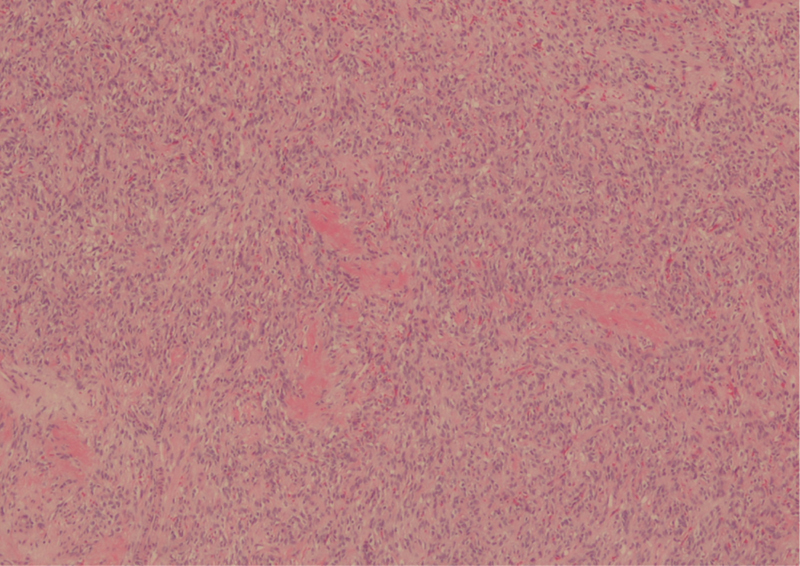
Intra-articular schwannoma. Nuclear palisading and verocay bodies.

A 54-year-old Caucasian man with a painful lump on his left leg and a medical history of arterial hypertension visited the orthopaedic department. He denied any history of trauma prior to symptom onset. His clinical examination revealed a firm mass in the proximal medial aspect of his left leg and a reduced range of extension. He experienced increased pain with passive and active dorsiflexion. Magnetic resonance imaging (MRI) of the leg was performed for further evaluation ( Fig. 1 ). A mass measuring 64 × 34 mm projecting a soft tissue in the medial part of the patellar tendon was noted in the infrapatellar space/fat pad ( Fig. 2 ). The mass was histologically evaluated using fine-needle biopsy; results revealed fusiform cell proliferation with low proliferative index but no necrotic areas ( Fig. 3 ). Immunological labeling of the cells demonstrated the presence of protein S100 and expression of CD34, indicating neurogenic origin ( Fig. 4 ). Surgical treatment was proposed and subsequently performed ( Fig. 5 ). The tumor was resected using the medial parapatellar approach ( Fig. 6 ). Histological examination confirmed the diagnosis of a schwannoma ( Fig. 7 ). The patient was followed-up in the outpatient clinic; he experienced complete recovery after 6 months without any limitation to mobilization and remains asymptomatic 3 years later.

## Discussion

Most solitary HFP tumors are benign. In 2011, Dean et al presented 19 patients with benign tumors of the intra-articular ganglia with pigmented villonodular synovitis as the most common diagnosis. 4 In 2013, Albergo et al presented 25 patients who were most commonly diagnosed with pigmented villonodular synovitis and hemangioma; two malignant tumors were reported, and none of those were diagnosed with schwannoma. 5 Despite its rarity, schwannoma should be considered in the differential diagnosis.

The present case report describes the discovery of an intra-articular benign tumor of the peripheral nerves. Schwannomas usually occur in the fourth and fifth decades of life and have a female predilection of 1.6:1. 6 Schwannomas are often asymptomatic and discovered incidentally owing to their slow growth. In the present case, the patient's pain corresponded with the progressive tumor growth. In the literature, solitary tumors have been reported in various locations; however, their occurrence in the lower limbs is rare. 7 To the best of our knowledge, only one case report has described an intra-articular schwannoma of the knee. 8

Evaluation of the patient's symptoms, history taking, and physical examination suggested the presence of a tumor. Microscopic analysis helped to confirm the diagnosis of a schwannoma. The patient's preoperative plain radiographs and MRI were assessed. Consequently, radical surgical excision was proposed and performed based on the patient's symptomatology.

Although radiography should be initially performed to rule out the possibility of malignant tumor, MRI remains the imaging technique of choice for accurate diagnosis. Regarding solitary tumors, they are frequently symptomatic, and excellent outcomes can be achieved using open excision, which ensures that solitary HFP tumors are completely removed. 4

## Conclusion

This is the second reported case of a larger intra-articular schwannoma of the knee since 1994. Considering the rarity of this tumor, the present report is a great addition to the limited literature.
